# Debrief it all: a tool for inclusion of Safety-II

**DOI:** 10.1186/s41077-021-00163-3

**Published:** 2021-03-29

**Authors:** Suzanne K. Bentley, Shannon McNamara, Michael Meguerdichian, Katie Walker, Mary Patterson, Komal Bajaj

**Affiliations:** 1 Departments of Emergency Medicine and Medical Education, Icahn School of Medicine at Mount Sinai, New York, NY USA; 2 Simulation Center at Elmhurst, NYC Health + Hospitals/Elmhurst, 7901 Broadway, Elmhurst, NY 11373 USA; 3 Simulation Center of NYC Health + Hospitals, 1400 Pelham Pkwy S, Bronx, New York, NY 10461 USA; 4 Department of Emergency Medicine, NYU Langone Health, 550 1st Ave, New York, NY 10016 USA; 5 Department of Emergency Medicine, NYC Health + Hospitals/Harlem, 506 Lenox Ave, New York, NY 10037 USA; 6 Department of Emergency Medicine, College of Medicine of the University of Florida, Gainesville, FL USA; 7 University of Florida Center for Experiential Learning and Simulation, 1104 Newell Dr, Gainesville, FL 32610 USA; 8 Department of Obstetrics & Gynecology, Albert Einstein College of Medicine, Bronx, New York, NY USA

**Keywords:** Safety-II, Debriefing, Simulation, Error, Patient safety

## Abstract

Safety science in healthcare has historically focused primarily on reducing risk and minimizing harm by learning everything possible from when things go wrong (Safety-I). Safety-II encourages the study of all events, including the routine and mundane, not only bad outcomes. While debriefing and learning from positive events is not uncommon or new to simulation, many common debriefing strategies are more focused on Safety-I. The lack of inclusion of Safety-II misses out on the powerful analysis of everyday work.

A debriefing tool highlighting Safety-II concepts was developed through expert consensus and piloting and is offered as a guide to encourage and facilitate inclusion of Safety-II analysis into debriefings. It allows for debriefing expansion from the focus on error analysis and “what went wrong” or “could have gone better” to now also capture valuable discussion of high yield Safety-II concepts such as capacities, adjustments, variation, and adaptation for successful operations in a complex system. Additionally, debriefing inclusive of Safety-II fosters increased debriefing overall by encouraging debriefing when “things go right”, not historically what is most commonly debriefed.

## Introduction

### Safety science

Safety science has been shifting focus. For decades, professionals in safety critical industries have focused primarily on reducing risk and minimizing harm by learning everything they can from when things go wrong [1, 2]. Despite tremendous investment in this strategy, the outcomes are disappointing [3]. Safety science continues to evolve and the limitations of a Safety-I only strategy are becoming clearer with growing imperative to expand our thinking and practices [4].

Healthcare is a safety critical industry [5]. The daily work of healthcare professionals contributes directly to life and death outcomes for patients. Healthcare is also a complex adaptive system [2, 6, 7]. Its elements are usually emergent and nonlinear, resulting in unexpected and variable outcomes. It is often difficult, if not impossible, to determine causality with complete certainty [1, 2, 6]. Knowing this, it is challenging to know how best to improve performance and limit harm in when linear models of cause-and-effect rarely apply. Furthermore, it is a challenge to account for the importance of adaptive capacity when defining good performance.

### Safety-I…and Safety-II…and Safety-III

**Fig. 1:**
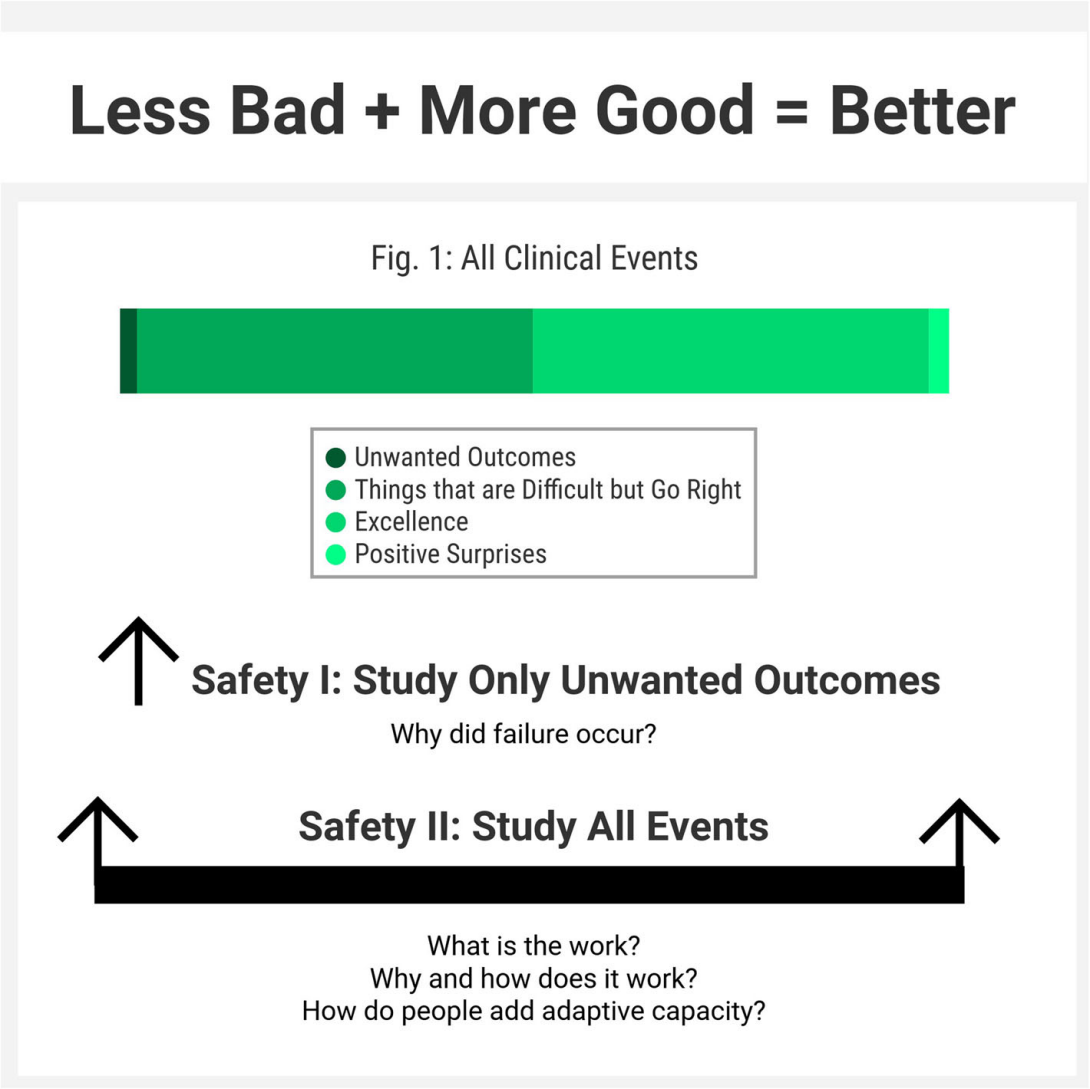
Safety-I vs. Safety-II study of clinical events

A not unfamiliar occurrence to many debriefers is asking an individual or a team to debrief and being met with a response such as “it went great, I don’t think we need to debrief, I wouldn’t have done anything differently.” This is a common example of a historically Safety-I focused mindset around debriefing and a prime opportunity to shift and expand focus. As stated by Sidney Dekker, “Safety is not about the absence of negatives. It is about the presence of capacities.” [8]. Safety-II offers a paradigm that expands on Safety-I to account for the presence of capacities, as well as the complexity and adaptivity of healthcare systems [1, 2, 4, 9]. Safety-II encourages us to study and debrief all events, including the routine and mundane, not only bad outcomes (Fig. 1). By examining everyday work as done, we must confront the reality that written policies and actual practice are often different. Examining work as done also shows the necessity of performance adjustments, variation, and adaptation for successful operations in a complex system. By studying all work and all outcomes, interconnectedness, dependencies, and patterns of systems behavior emerge over many incidents. This information is tremendously powerful and often underutilized in debriefings and in general.

Recently, drawing from her experience primarily in aerospace and defense, Leveson has introduced the concept of “Safety-III”, suggesting a safety management principle that “concentrates on preventing hazards and losses, but does learn from accidents, incidents, and audits of how system is performing.” [10]. We believe that the notion of applying debriefings to both the positive and negative elements of an event is consistent with Leveson’s Safety-III approach.

### Safety and role of debriefing

Debriefing after simulated or real clinical events is a powerful tool to capture the knowledge and adaptations of frontline healthcare workers. Routine debriefing also facilitates understanding of system resources and constraints [11]. Debriefing methods have been largely adopted and adapted from aviation and psychology and there are many models of both post-simulation and clinical event debriefing [11–19].

### Need for debriefing inclusive of Safety-II

Debriefing is a growing practice in healthcare, conducted after planned simulations and high stakes and team events (e.g., cardiac arrest resuscitation, postpartum hemorrhage, trauma care) or unfavorable outcomes and has been demonstrated to improve performance [20] and clinical outcomes [12, 21–23]. While debriefing and learning from “positive events” is not uncommon or new to simulation, both simulation and clinical event debriefing programs are often more focused on Safety-I debriefing [20, 23].

Multiple studies support that there is no one correct way to debrief and many advocate for utilizing blended approaches and strategies [11, 24–26]. Though several frameworks include analysis of positive performance, there is often limited discussion of the Safety-II concepts such as adaptation, resource utilization, constraints, variability, and work as done [27]. Since the impact of a Safety-I approach is limited, how can clinicians incorporate and bolster Safety-II thinking into the practice of debriefing to improve systems level performance? How can we go beyond learning from failure to debriefing for learning from success [27]? A mindset constricted to Safety-I may not anticipate the value of debriefing when things go well, despite the rich learning potential.

### Vignette exploring Safety-II

The following case vignette highlights the value in analyzing when things go right and how to ensure they go right as frequently as possible: An in situ simulation in the pediatric emergency department involving critical care resuscitation with intubation went smoothly with seemingly maximized teamwork and communication, quick equipment retrieval, and first pass success with intubation.

The team leader’s response to request to debrief was “It went great, I don’t think we need to, I wouldn’t have done anything differently.” This reaction was not surprising but rather reflects an anecdotally common perception that debriefings are reserved for “bad outcomes” and “fixing things.” The team appeared genuinely surprised (and fairly apprehensive) of the persistent request to still debrief. Over the next 5 min, a robust discussion unfolded. No one was “sure” why the case went so smoothly. SB posed the question “are there strategies you used in this case or in your normal work with intubation in order to be more efficient or effective?” The resident thanked the nurse for having all of the airway equipment laid out for him and asked the nurse how he has become so efficient with the airway equipment. The resident noted a recent experience in which he struggled to locate a specific endotracheal tube size during a previous pediatric intubation. The nurse, shyly at first, explained that he, and others in this area, finds the airway cart to be very confusing so they actually stock backup endotracheal tubes and blades in a medication room near the resuscitation room. For efficiency, he skipped the airway cart entirely and ran to the medication room to quickly obtain the proper equipment. This reflection uncovered a workaround representing “work as done” that created better performance and could inform reliable performance generalizable to other areas.

The successful workaround was shared with quality leadership and location and layout of the airway carts were changed with this single insight of “work as done” vs. “work as imagined” captured from the nurse during this debriefing. This example of the value of Safety-II and routinely analyzing when things go right highlights how application of Safety-II concepts during debriefing can broaden discussions, capture high yield analysis and improvements that may otherwise not be discussed, and create a broader scope of change applicable to other units who may have similar masked challenges.

Given the paucity of literature and recommendations for how best to debrief for Safety-II concepts (in addition to Safety-I concepts), valuable opportunities for discussion, learning, and systems improvement may be missed. The objective of this pilot was to utilize expert consensus to create a tool for debriefing with a Safety-II focus, including highlighting key Safety-II concepts and provision of sample phraseology.

## Methods

In order to provide specific Safety-II related language for each phase of a debriefing including (1) setting the scene, (2) clarifying facts, (3) analysis, and (4) summary, a tool was developed through expert consensus. The language inclusive of a Safety-II focus was iteratively drafted by the simulation expert authors of this manuscript, who have combined greater than 50 years of simulation/debriefing experience using Safety-II language in practice. The tool was introduced to 2 pilot groups: one group of 3 senior simulationists and 3 simulation fellows, all clinicians from different disciplines, and the 2nd group was comprised of 4 senior simulationists. Both groups were surveyed after using the tool following a variety of case types and varied interprofessional learner types, both in situ and in the simulation center for feedback. Quantitative feedback was obtained via survey with Likert-style questions related to overall impression, readability, and anticipated use of the tool. Qualitative feedback was captured during focused discussion with both groups on overall perception of the tool, comments, and any recommended changes to content or language. Final edits were incorporated into tool provided in Table 1.

**Table 1:** Safety-II debriefing tool

Debriefing phase and goal	Safety-II concept(s) highlighted	Sample language/phrases
Debriefing introduction/setting the scene	• Safety-II expands on Safety-I (study of failures) to analyze the complexity and adaptability of the system and capitalize on good performance. • “Safety is not about the absence of negatives; it is about the presence of capacities” [8].	• Let’s take a look at how our work really operates, including the systems and relationships that support us. • We’ll also discuss the challenges we may encounter and how we adapt to overcome those challenges. • How we adapt in different circumstances offers insights into why we succeed. • Understanding how things work and why things go right helps us improve. • Our goal is to collaboratively discuss this case, the outcomes, and the performance aspects that went well and why, so we may better understand and capitalize on them in the future. • In addition, we will discuss opportunities for improvement.
Case summary/description	Value of understanding normal workflow (work as done vs. work as imagined)	Can you please share the facts/short summary of the case?
Analysis	• How does the work actually work? • Variability • Adaptability • Flexibility • Workarounds • Near misses and harm mitigation strategies • Reproducing success • Leveled hierarchies/ability to share concerns • What conditions make success more likely? What conditions make success more difficult?	Let’s focus on what went well: • Why did *X* go so well in this case? How can we ensure this happens again this way in the future? • How did people adapt to overcome challenges in this case? What behaviors facilitated good performance? • What resources enabled good performance? • How does this work usually happen? Are the behaviors and/or resources reliably available/performed? • Are there strategies that were used in this case or that you use in your normal work to be more efficient or more effective? • How has this played out during a similar clinical situation? Are there examples of cases like today’s when it didn’t go well? What is the difference between that case and today? • How do we ensure reliability of available resources and encourage useful behaviors? Let’s now explore what could be done differently or improved: • Let’s specifically discuss *X* that could have been done differently this time. Has it gone right before? Why has it gone right/differently other times but not during today’s case? • Were there any near misses? If so, how did the team adapt to prevent harm from occurring? (e.g., *X* event? Mutual support between Nurse *X* and Dr. *Y* prevented medication being administered into an IV line that Nurse *X* noticed was infiltrated) • Were there systems challenges encountered that made this case more difficult than it needed to be? How could those systems improve to support your work in the future?
Summary/take home points	• Reproducing success • Identifying opportunities for systems improvement	What occurred in this case that we want to continue in the future? e.g., What is needed to ensure this happens reliably again in the future? How can each of us help to make this happen?

Additionally, use of the tool was introduced for piloting into 1 author’s (SB) in situ cardiac arrest simulation program. Analysis of frequency of use by facilitators provided with the tool and change in type and number of topics discussed during debriefing were recorded, in comparison to type and number of topics recorded without this tool.

## Results

Through searching the literature, expert consensus, and iterative revision based on feedback and piloting, Table 1 represents a tool consisting of specific strategies and sample language and phrases that may be employed to facilitate debriefings incorporating Safety-II concepts. Debriefing phases emphasizing Safety-II concepts included are phases of “setting the stage,” “summarizing the case,” “analysis”, and “summary/take home points.” Safety-II concepts are provided matched with debriefing phase and sample language/phrases to elicit/explore each concept.

Pilot group participants surveyed (*N* = 10) responded with 100% noting “strongly agree” or “agree” to all questions regarding utility and usability of the tool (Table 2). Free text survey comments included “Overall, this tool added much value to depth of our debriefing”, “This tool is readable as phrased and formatted”, “The questions were understandable to a variety of learners types and of different levels”, “The phrases are clearly linked to concepts indicated in the chart and helped me incorporate them for discussion”, and 100% noted “strongly agree” to “I would likely include this in future debriefings.”

**Table 2:** Survey responses of perceived utility and usability of the Safety-II debriefing tool

	% Strongly agree	% Agree
Overall, this tool would add/added value in my debriefings.	100	0
This tool is readable as phrased and formatted.	83	17
The questions will be/were understandable to a variety of learners.	83	17
The phrases are clearly linked to concepts indicated in the chart.	100	0
I would likely include this in future debriefings.	100	0

Comments made during facilitated pilot group discussion with the same participants included “this will be so useful to probe deeper during a ‘smooth’ case”, “I will definitely use this again, it helped me expand on the ‘why did it go right’ question I always try to ask”, and “I fumble to encourage learners to think about the good things and value and learning from the good, this will definitely help.” When asked specifically about post-event clinical debriefing, “do you feel this tool might change the types of cases you choose to debrief”, respondents voiced perceived value in using this for any case type and the potential to increase the number of debriefings done. One noted, “I see how this language could be used for any case, even if a great outcome, and I think having the language examples would help me better frame that discussion.”

Additionally, pilot of inclusion of the tool during 3 debriefings of 1 author’s recurring in situ cardiac arrest simulations revealed increased number of types and overall numbers of topics discussed (as recorded on post-debriefing data form and coded into topic areas) from an average of 21 in the 3 debriefings that used the tool compared to an average of 14 in the 3 preceding in situ cardiac arrest simulations without availability of this tool. New topics recorded included an example such as “mutual support was observed. A senior resident stepped in to assist an intern with intraosseous drill placement when initial drill assembly was incorrect” (preventing intern from continuing with incorrect set up and potentially injuring himself or patient). This led to a productive discussion of why the resident was able to seamlessly step in and assist, and how this varied from other cases. The presence of a senior resident was the specific resource available that allowed for this adaptation. Other examples were then elicited and discussed of times during which mutual support for procedure completion occurred or would have provided benefit, if it had.

Another example was “discussed observation of calling respiratory therapy directly versus relying on paging system”. This led to the identification of a possible issue with respiratory therapy pagers. Most notably, however, it led to robust and generalized discussion of use of this workaround and other workarounds. Additional adjustments necessary to ensure work goes consistently well were also discussed. This highlighted an example of “work as done” (direct call) vs. “work as imagined” (paging via hospital operator).

All debriefing facilitators (*N* = 4) involved in the pilot cases noted overall satisfaction with inclusion of the tool. Comments included “it was pretty easy to use these phrases”, “I’m glad I had the actual language to ask about the concept I saw happen”, and “this helped me reframe the group when the case was quick and seemingly close to perfect.” Anecdotally, one author reported “there seems to be an interesting phenomenon that often appears in which it seems harder for some participants to discuss things that went well because they are perhaps so primed to seek out the error or what went wrong, however, I conversely also feel that this tool led to more participants sharing ideas and getting excited because it expanded discussion beyond specifics of just the one case.”

## Discussion

Approaches to debriefing after simulated or clinical events are evolving. Current literature suggests that explicit tools for debriefing inclusive of a Safety-II focus are rarely included. Framed as an expansion of previous debriefing theories, we created a tool via expert consensus to link key Safety-II concepts with sample phraseology to include in debriefings. This allows for an expansion from the focus on analysis of errors and “what went wrong” or “could have gone better” to also capture valuable discussion of high yield Safety-II concepts such as discussion of everyday work. Additionally, it encourages increased event debriefing overall by providing a tool to debrief when “things go right.”

Initial feedback from pilot groups indicates that they find the tool feasible and acceptable for use. Analysis of the breadth of topics covered during debriefings that included Safety-II prompts further suggests that this Safety-II debriefing tool is an initial step in broadening debriefing objectives and discussions. In our experience, we encourage debriefers to use the tool in all debriefings (to capture of Safety-II concepts even when there is an adverse outcome).

Next steps include implementation of this tool into educational debriefing programs for additional feedback, revision, and validation. In addition, we plan specific piloting of its use in clinical post-event debriefing programs. We will also study its use and impact on numbers and types of debriefings held and debriefing feedback outcomes when the tool is added to traditional debriefing methods. We believe the tool will expand the breadth of debriefing conversations and foster debriefing “all” events, regardless of outcome.

## Data Availability

Data sharing is not applicable to this article as no datasets were generated or analyzed during the current study.
